# Aquatic and Terrestrial Environmental Correlates of Eurasian Water Shrew (*Neomys fodiens*) Habitat Use in the Lesser Khingan Mountains

**DOI:** 10.3390/biology15181674

**Published:** 2026-09-21

**Authors:** Yao Zhao, Haoying Bian, Zhuo Wang, Zhitao Liu, Juncheng Li, Peng Liu

**Affiliations:** College of Life Science and Technology, Harbin Normal University, Harbin 150025, China; bianhaoying@163.com (H.B.); tatumao@126.com (Z.W.); zhitaoharbin1972@163.com (Z.L.); donghexiang2@163.com (J.L.)

**Keywords:** *Neomys fodiens*, habitat use, semi-aquatic mammal, aquatic–terrestrial interface, multimodel inference, Lesser Khingan Mountains

## Abstract

Understanding habitat selection is essential for predicting species distributions and developing effective conservation strategies, particularly for semi-aquatic mammals. However, habitat requirements of the Eurasian water shrew (*Neomys fodiens*, EWS) remain poorly understood in northeastern China. We investigated habitat use by comparing 44 used and 44 control plots in the Lesser Khingan Mountains during winter and spring. Aquatic and terrestrial environmental characteristics were evaluated separately using generalized linear models and multimodel inference, and supported predictors were subsequently combined in an integrated model. Habitat use decreased significantly with increasing distance from water (β = −0.333, *p* = 0.008), was higher in still-water than in fast-flowing habitats (β = 1.728, *p* = 0.04), and was substantially lower at upper-slope than at lower-slope sites (β = −2.712, *p* = 0.02). The integrated model received stronger support (AICc = 101.62) than the aquatic (AICc = 106.21) and terrestrial (AICc = 110.50) models and showed the highest discriminatory performance (AUC = 0.853). This advantage was retained under repeated stratified 10-fold cross-validation, with a mean AUC of 0.786 ± 0.013. These findings indicate that EWS habitat use is better characterized by considering aquatic and terrestrial environmental characteristics jointly rather than separately and highlight the importance of the aquatic–terrestrial interface for habitat assessment and conservation of this semi-aquatic species in northeastern China.

## Simple Summary

The Eurasian water shrew (*Neomys fodiens*) is a semi-aquatic mammal, but its habitat requirements remain poorly understood in northeastern China. We investigated habitat use by this species in the Lesser Khingan Mountains and found that habitat use was associated with proximity to water, water velocity, and slope position. A model combining aquatic and terrestrial environmental characteristics performed better than models considering either habitat component separately. These findings highlight the importance of considering the aquatic–terrestrial interface when assessing and conserving habitats for this semi-aquatic species.

## 1. Introduction

Habitat selection is a fundamental ecological process through which wildlife respond to environmental heterogeneity and meet their requirements for survival and reproduction [1]. By selecting suitable habitats, individuals can maximize resource acquisition, reduce predation risk, and maintain physiological performance under changing environmental conditions. Consequently, habitat selection influences population dynamics, species distributions, and ecological adaptation, and has become a key component of wildlife conservation and habitat management [2]. Semi-aquatic mammals represent a unique ecological group occupying the transition zone between aquatic and terrestrial ecosystems. Unlike fully terrestrial or fully aquatic species, semi-aquatic mammals must simultaneously respond to environmental conditions in both habitat types [3,4]. Aquatic habitats influence prey availability, hydrological conditions, and the energetic costs of swimming, whereas terrestrial habitats provide refuges, nesting sites, and opportunities for predator avoidance [5]. Evaluating the associations of these aquatic and terrestrial environmental characteristics with habitat use is particularly important for species that occur at low densities or are difficult to detect, for which ecological information is often limited.

The forest ecosystems of northeastern China, including the Greater and Lesser Khingan Mountains, represent an important natural forest region of China [6]. The Lesser Khingan Mountains are characterized by extensive river networks, well-developed riparian habitats, and pronounced seasonal climatic variation [7,8]. Long, cold winters and short, cool summers are accompanied by marked seasonal changes in temperature, hydrological conditions, snow cover, and vegetation structure [9]. These environmental conditions can impose substantial energetic constraints on small-bodied endotherms because of increased thermoregulatory demands and seasonal fluctuations in resource availability [10]. At the same time, the heterogeneous mosaic of forests, wetlands, streams, and riparian habitats supports diverse wildlife communities and contributes to the regional conservation value of the Greater and Lesser Khingan Mountains [11]. Together, pronounced seasonal climatic variation, extensive river networks, and heterogeneous riparian environments create substantial environmental heterogeneity across the aquatic–terrestrial interface.

Such environmental heterogeneity provides an appropriate system for examining how aquatic and terrestrial habitat characteristics are associated with habitat use by a semi-aquatic mammal. The Eurasian water shrew (*Neomys fodiens*, EWS) is one of the smallest semi-aquatic insectivorous mammals and exhibits one of the highest mass-specific metabolic rates among mammals [12]. To sustain its exceptionally high energetic demands, individuals must forage almost continuously throughout the day [13]. Their diet is composed predominantly of aquatic macroinvertebrates, making stream margins and shallow-water habitats the primary foraging environments [14]. Previous studies have shown that its occurrence can be associated with characteristics of both the aquatic environment and surrounding riparian habitat. In southeastern England, water velocity, water depth, bank incline, and bank-side vegetation were associated with EWS occurrence [15], whereas another study identified freshwater crustaceans and riparian vegetation characteristics as important correlates of site occupancy [16]. More recently, radio tracking combined with LiDAR-based habitat characterization showed that fine-scale habitat use was associated with vegetation structure and proximity to water, with the probability of EWS occurrence decreasing as distance from water increased [17].

Despite these advances, how aquatic and terrestrial environmental characteristics jointly characterize EWS habitat use remains insufficiently resolved. Previous studies have identified individual habitat characteristics associated with occurrence or fine-scale habitat use, but aquatic and terrestrial characteristics have rarely been explicitly compared within a common modeling framework [18,19]. This distinction is important because EWS occupies the aquatic–terrestrial interface, where characteristics of the water body and surrounding terrestrial environment may contribute simultaneously to habitat use. Moreover, most detailed studies of EWS habitat relationships have been conducted in Europe [15,16,17], whereas ecological information from northeastern China remains limited. This limits our understanding of whether habitat associations identified elsewhere also characterize EWS populations occurring in cold-temperate forest environments.

In this study, we evaluated habitat use by EWS in the Lesser Khingan Mountains of northeastern China using an integrated aquatic–terrestrial framework. Specifically, we aimed to (1) identify aquatic environmental characteristics associated with EWS habitat use; (2) identify terrestrial environmental characteristics associated with EWS habitat use; and (3) determine whether an integrated model combining aquatic and terrestrial characteristics provides stronger model support and discriminatory performance than models representing either habitat component separately. By explicitly comparing aquatic, terrestrial, and integrated models, this study evaluates whether combining environmental characteristics from both habitat components improves model support and discriminatory performance for EWS habitat use in northeastern China.

## 2. Materials and Methods

### 2.1. Study Area

**Figure 1:**
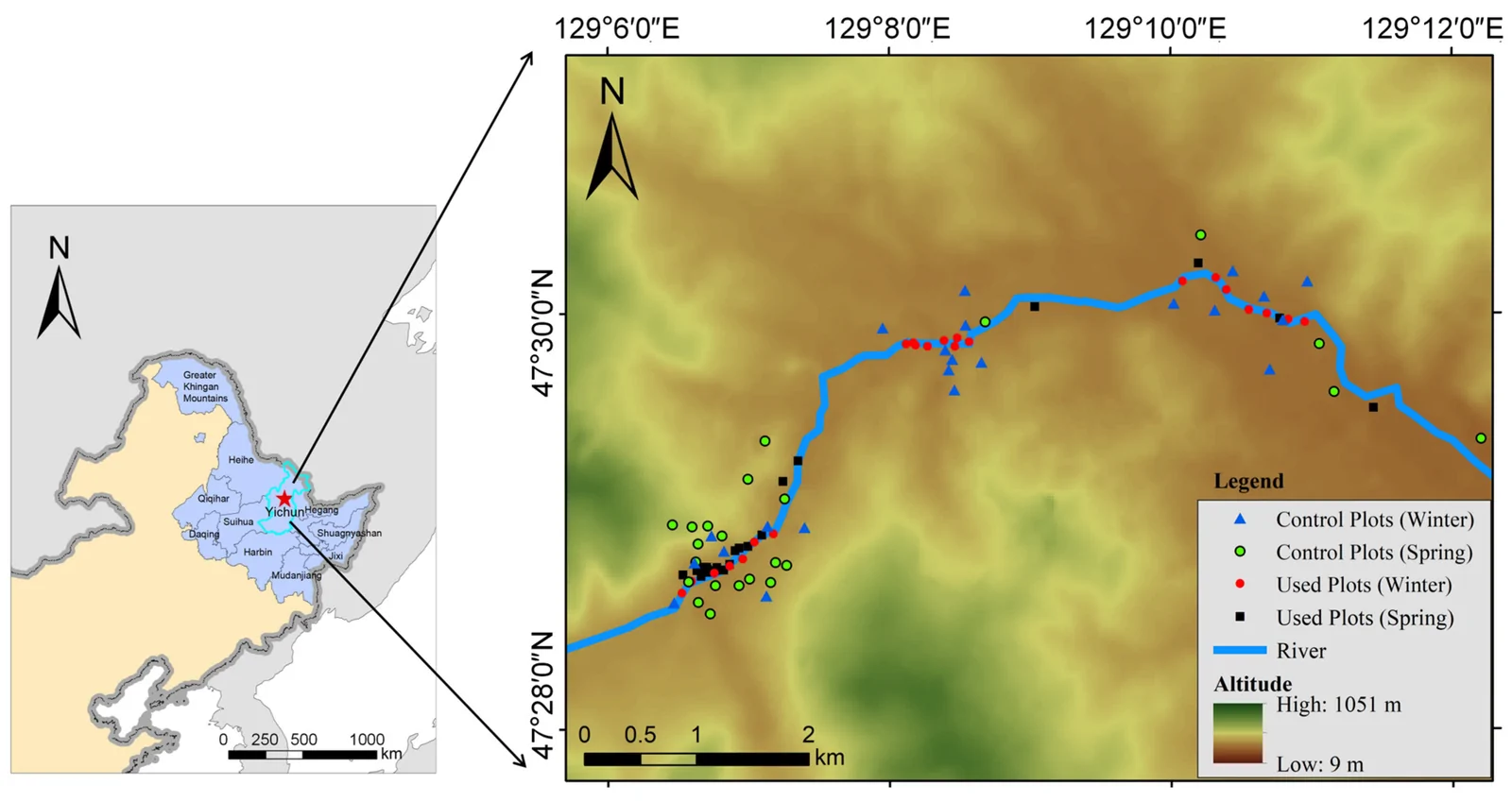
Location of the study area and sampling plots for Eurasian water shrew (*Neomys fodiens*, EWS) in the Lesser Khingan Mountains, northeastern China.

This study was conducted in the Lesser Khingan Mountains region (47°31′–47°47′ N, 129°09′–129°27′ E) located in Yichun City, Heilongjiang Province. Field surveys were conducted during two seasonal sampling periods: winter (December 2025–January 2026) and spring (March–May 2026). Sampling plots were progressively established and surveyed throughout each seasonal survey period. The region is located within the cold-temperate forest zone and is characterized by extensive river networks, well-preserved forest ecosystems, and diverse riparian habitats [7,8]. The climate is strongly seasonal, with long, cold winters and short, relatively warm summers. Mean annual temperature ranges from approximately −1 to 2 °C, and annual precipitation ranges from 500 to 700 mm, with most precipitation occurring during the growing season [20]. The forests of the Lesser Khingan Mountains show substantial spatial heterogeneity and are characterized by mixed broad-leaved and coniferous communities, including larch and European birch (*Betula platyphylla*) [21]. These extensive forest ecosystems provide habitat for diverse wildlife, including the Siberian tiger (*Panthera tigris*) and Siberian musk deer (*Moschus moschiferus*) [22,23]. Riverbed substrates, riparian vegetation, and local microhabitat conditions are also highly heterogeneous. Together with the well-developed drainage network and extensive wetlands, these features create a complex landscape of aquatic and terrestrial habitats (Figure 1).

### 2.2. Data Collection

**Figure 2:**
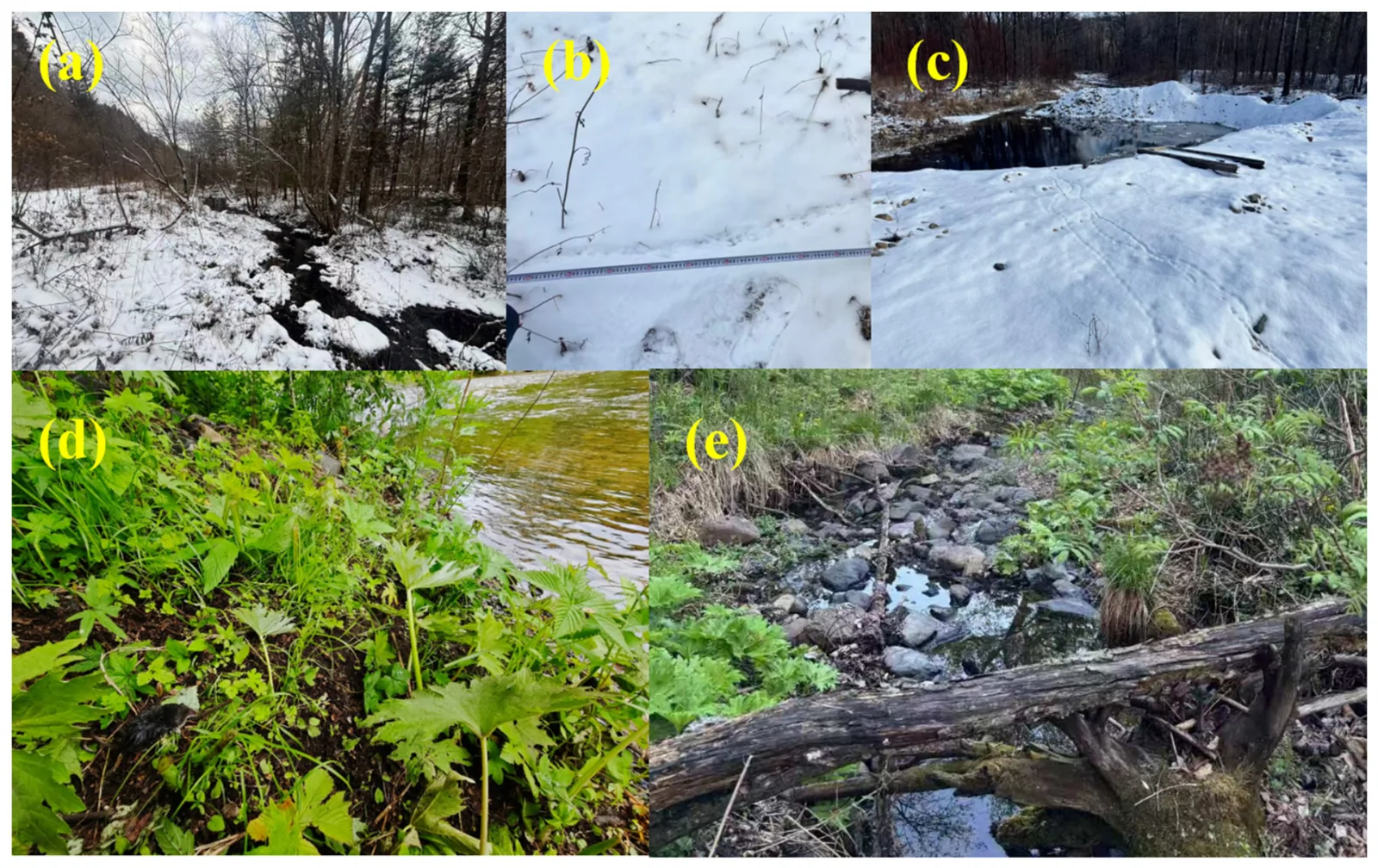
Field evidence of Eurasian water shrew (*Neomys fodiens*) activity recorded during field surveys in the Lesser Khingan Mountains. (**a**) Habitat where EWS activity was detected during the winter survey; (**b**) EWS footprints recorded during field surveys; (**c**) EWS footprints recorded in snow; (**d**) EWS observed along the shoreline during the spring survey; and (**e**) habitat associated with EWS activity.

Because EWSs are rare, elusive, and difficult to observe directly, locations of EWS activity were identified through field surveys based on direct observations and diagnostic signs, including footprints, feces, burrow entrances, and other field traces. Identification was based on multiple diagnostic characteristics to reduce the risk of confusion with sympatric small mammals. Only locations with diagnostic evidence consistent with EWS activity were classified as used locations. The specific type of diagnostic evidence associated with each used plot was not systematically recorded. Compared with sympatric rodents, EWS footprints are characterized by five distinct toes on both forefeet and hind feet, slender digit impressions, visible claw marks, and relatively narrow track patterns (Figure 2).

When evidence of EWS activity was detected, a 10 × 10 m used plot centered on the detected trace was established to characterize habitat conditions associated with EWS use. Control plots of the same size were established during the same seasonal survey in the same general riparian environment at locations where no diagnostic evidence of EWS activity was detected. The field sampling used a 1:1 used-to-control allocation, resulting in equal numbers of used and control plots within each season. Control plots therefore represented locations where no diagnostic evidence of EWS use was detected during the corresponding field survey and were not interpreted as confirmed absence sites. The same field team and standardized survey procedure were used throughout the study; however, plot-level search duration was not recorded quantitatively. Spring and winter plots represented season-specific sampling locations rather than repeated measurements of the same fixed plots. All habitat variables were measured by the same field team following a standardized protocol to maintain consistency among sampling plots and seasons.

Multiple surveys conducted during spring and winter yielded 88 habitat records in total, comprising 44 used and 44 control plots, with 22 used and 22 control plots in each season (Figure 1). Water temperature was recorded using iButton data logger (DS1921, Dallas Semiconductor, Dallas, TX, USA), while microhabitat temperature was monitored using HOBO data loggers (MX1104, Onset Computer Corporation, Bourne, MA, USA). Water and microhabitat temperatures were recorded at 1 h intervals for three consecutive days at each sampling plot. The two temperature loggers were deployed simultaneously at each plot and therefore covered the same monitoring period. For each plot, the mean and standard deviation were calculated from all hourly records collected during the three-day monitoring period and were subsequently used as plot-level temperature variables in the statistical analyses. A consistent three-day monitoring period was applied to all sampling plots to standardize the duration of temperature measurements among plots.

### 2.3. Environmental Variables

Environmental variables were classified into two ecological components: aquatic and terrestrial variables. Water-temperature variables were included in the aquatic component, whereas terrestrial microhabitat-temperature variables were included in the terrestrial component (Table 1). These variables were selected to represent complementary aspects of habitat suitability, with aquatic variables reflecting potential effects of water availability and aquatic conditions, and terrestrial variables representing refuge, thermal buffering, and landscape characteristics [24].

Aquatic environmental variables included distance to water, water velocity, water depth, water width and water temperature. Water velocity was visually classified in the field as fast, slow, or still based on the overall flow conditions of the water body adjacent to each 10 × 10 m sampling plot. The classification was based on visual assessment rather than instrumental measurements of current velocity: fast, characterized by clearly visible continuous flow and surface turbulence; slow, characterized by continuous but weak water movement with little surface turbulence; and still, characterized by no visually detectable surface flow at the time of measurement. The same field team applied the same classification procedure during both spring and winter surveys to maintain consistency among sampling plots and seasons. No formal inter- or intra-observer repeatability assessment was conducted for the visual classification. Accordingly, the fast, slow, and still categories should be interpreted as standardized qualitative field classifications rather than objective quantitative measurements of current velocity. The surveyed water bodies remained unfrozen during the winter sampling period. Therefore, winter classifications were based on directly observable water-flow conditions rather than surface-ice conditions. Distance to water was measured as the shortest distance between the plot center and the nearest stream channel. Water depth was measured directly using a graduated ruler. Water width was measured at three representative cross-sections of the water body adjacent to each sampling plot, with width defined as the distance between the two water edges. The mean of the three measurements was used to represent water width for each plot.

Terrestrial environmental variables included altitude, slope, aspect, slope position, human disturbance, vegetation cover, and vegetation type. Vegetation type was classified according to the dominant vegetation structure surrounding each sampling plot as mixed forest, riparian vegetation, or shrub. Riparian vegetation referred to vegetation occurring directly along the stream margin and dominated by streamside herbaceous vegetation, whereas shrub habitat was characterized by dominant woody shrub cover and mixed forest by a tree-dominated vegetation structure. Human disturbance was categorized according to the intensity of anthropogenic influence, including low, moderate, and high disturbance levels. Environmental variables were classified into aquatic and terrestrial components according to their ecological context.

### 2.4. Statistical Analysis

Prior to analysis, continuous environmental variables were assessed for normality using the Shapiro–Wilk test. Because the variables generally deviated from normality, differences between used and control plots and between seasons were evaluated using Wilcoxon rank-sum tests. Associations between categorical habitat variables and habitat use were assessed using chi-square tests. Because the original plot-level pairing identifiers were unavailable, the used–control univariate comparisons were also conducted as unpaired exploratory analyses and should not be interpreted as pair-adjusted tests. Pairwise correlations among continuous environmental variables were examined using Spearman’s rank correlation coefficients [25]. Multicollinearity among predictors was evaluated using generalized variance inflation factors (GVIFs), with GVIF^(1/(2×Df))^ used for multi-level categorical predictors. Habitat use was analyzed using generalized linear models (GLMs) with a binomial error distribution and logit link function, with habitat status (used vs. control) as the response variable. Here, “control” denotes the absence of detected diagnostic evidence during the field survey rather than confirmed species absence. Candidate environmental predictors were assigned a priori to aquatic and terrestrial groups. Aquatic predictors included Mean WaterTemp, SD WaterTemp, DisWater, Velocity, Depth, and Width. The initial terrestrial predictor set included Mean MicroTemp, SD MicroTemp, altitude, slope, slope aspect, SP, human disturbance, substrate type, soil, cover, and vegetation type. Following the collinearity assessment described below, Mean MicroTemp was excluded from subsequent candidate models because of its strong correlation with Mean WaterTemp. Season was retained as a temporal covariate in all candidate models.

Although field sampling followed a 1:1 used-to-control allocation within each season, the archived analytical dataset did not retain a reliable plot-level pairing identifier. Consequently, the original field pairing could not be reconstructed unambiguously. A conditional logistic regression or a model including a pair-specific random effect would therefore require retrospectively assigning pairs that are not supported by the archived data. We considered such reconstruction inappropriate because it could introduce an artificial dependence structure. We therefore retained the binomial GLM as an exploratory plot-level analysis. Importantly, the spatial analyses described below evaluate geographic dependence among sampling locations but do not reproduce or statistically account for the unrecoverable original used–control pairing. Accordingly, coefficient estimates, standard errors, and associated *p* values from the binomial GLMs should be interpreted cautiously as exploratory plot-scale habitat-use associations rather than pair-adjusted effects.

Separate global binomial GLMs were constructed for the aquatic and terrestrial predictor groups using predictors retained following the collinearity assessment. For each habitat component, all possible subsets of the environmental predictors in the corresponding global model were generated using the dredge function in the MuMIn package, while Season was retained as an a priori temporal covariate in every candidate model. Candidate models were ranked using Akaike’s information criterion corrected for small sample size (AICc). Models with ΔAICc < 2 were considered to have substantial support and were included in model averaging [26]. When multiple models met this criterion, full model averaging was performed across the supported model set. Model-averaged coefficients, adjusted standard errors, 95% confidence intervals, odds ratios (ORs), and summed Akaike weights (Σw) were used to quantify effect sizes, uncertainty, and relative predictor support. Predictors receiving strong support in the component-specific analyses (Σw ≥ 0.80) were carried forward to construct a parsimonious integrated aquatic–terrestrial model, with season retained as an a priori temporal covariate. The Σw ≥ 0.80 criterion was used as an operational threshold for model construction rather than as a formal significance criterion. Because predictor inclusion in the integrated model depended on the preceding component-specific multimodel analyses, this two-stage procedure does not fully propagate model-selection uncertainty from the initial screening step into the integrated-model estimates. The integrated model was therefore treated as an exploratory synthesis of strongly supported predictors rather than as a fully prespecified confirmatory model. Model support among the aquatic, terrestrial, and integrated models was compared using AICc and ΔAICc. For the aquatic and terrestrial components, the top-ranked AICc model was used for model-level performance comparisons, whereas model-averaged estimates were used for inference on individual predictors.

Model discrimination was assessed using receiver operating characteristic (ROC) curves and the area under the curve (AUC). Ninety-five percent confidence intervals for apparent AUC were estimated using 2000 stratified bootstrap replicates. Out-of-sample discrimination of the selected model structures was evaluated using stratified 10-fold cross-validation repeated 100 times [27]. In each repetition, models were refitted on nine folds and predictions were generated for the held-out fold. Cross-validated AUC was calculated from the pooled out-of-fold predictions and summarized as the mean ± SD across repetitions. To assess whether predictive performance was sensitive to spatial leakage between training and test data, we additionally performed five-fold spatially grouped cross-validation based on the geographic coordinates of the sampling plots. Plots were grouped according to spatial proximity so that geographically clustered observations were retained within the same validation group. Each spatial group was withheld in turn, the integrated model was refitted using the remaining groups, and predictions were generated for the held-out observations. Out-of-fold predictions from all five spatial groups were pooled to calculate AUC. This analysis was used as a spatial sensitivity assessment complementary to the repeated stratified 10-fold cross-validation. Because the original plot-level pairing identifier was unavailable, this spatial grouping could not guarantee that the original used–control pairs were assigned to the same validation fold. The spatially grouped cross-validation should therefore be interpreted as a sensitivity analysis for geographic leakage rather than as a pair-preserving validation procedure.

Potential spatial dependence in the final integrated model was evaluated by testing residual spatial autocorrelation using Moran’s I. Row-standardized k-nearest-neighbor spatial-weight matrices were constructed from the geographic coordinates of the 88 sampling plots. Because the sampling locations were distributed along spatially structured riparian habitats, residual spatial dependence was evaluated across three neighborhood definitions (k = 4, 6, and 8) rather than relying on a single spatial-weight specification. These settings represent progressively broader local neighborhoods in terms of the number of geographically nearest sampling plots considered, but do not correspond to fixed geographic distances because the physical distance to the kth nearest neighbor varies among sampling locations. Statistical significance was evaluated using 9999 Monte Carlo permutations. To evaluate whether inference from the integrated GLM was sensitive to explicitly modeled spatial structure, we additionally fitted a spatial generalized additive model (GAM) with a binomial error distribution and logit link, using the same environmental predictors as the integrated GLM together with a low-complexity two-dimensional smooth of projected geographic coordinates [s(X, Y), k = 5]. Here, k = 5 denotes the basis dimension of the spatial smooth and is distinct from the number of nearest neighbors used in the Moran’s I analyses. The spatial GAM was evaluated alongside the integrated GLM to determine whether the direction and statistical support of the principal habitat-use associations changed after accounting for geographic spatial structure. Residual spatial autocorrelation was subsequently reassessed for the spatial GAM using the same Moran’s I procedure.

Model convergence and influential observations were additionally examined for the final integrated GLM using standard regression diagnostics. All statistical analyses were conducted in R (v 4.4.1, R Core Team, Vienna, Austria), with *p* < 0.05 considered statistically significant. Spatial data processing was performed using ArcGIS (v 10.8, Esri, Redlands, CA, USA).

## 3. Results

### 3.1. Differences in Environmental Characteristics Between Used and Control Plots

Environmental variables showed considerable variation across the study area, reflecting pronounced heterogeneity in stream morphology, microclimate, topography, and riparian vegetation (Table S1). Univariate analyses revealed significant differences between used and control plots for several environmental variables (Table 2). Among the continuous variables, SD MicroTemp, DisWater, and Cover differed between used and control plots (*p* < 0.05). Among the categorical variables, the distributions of Velocity (χ^2^ = 8.54, df = 2, *p* < 0.05), SP (χ^2^ = 12.91, df = 2, *p* < 0.001), and Vegetation type (χ^2^ = 7.06, df = 2, *p* < 0.05) differed between used and control plots. In contrast, Mean WaterTemp, altitude, slope, depth, width, soil, slope aspect, substrate type, and human showed no significant differences between used and control plots. Among continuous variables, Mean WaterTemp, water temperature variability, Mean MicroTemp, SD MicroTemp, soil, and cover differed significantly between spring and winter sampling sites (Table 3). Among categorical variables, Human categories differed significantly between seasons (χ^2^ = 6.51, df = 2, *p* < 0.05). In contrast, altitude, slope, DisWater, Depth, Width, aspect type, SP, Velocity, substrate type, and vegetation type showed no significant seasonal differences.

### 3.2. Correlations Among Environmental Variables

**Figure 3:**
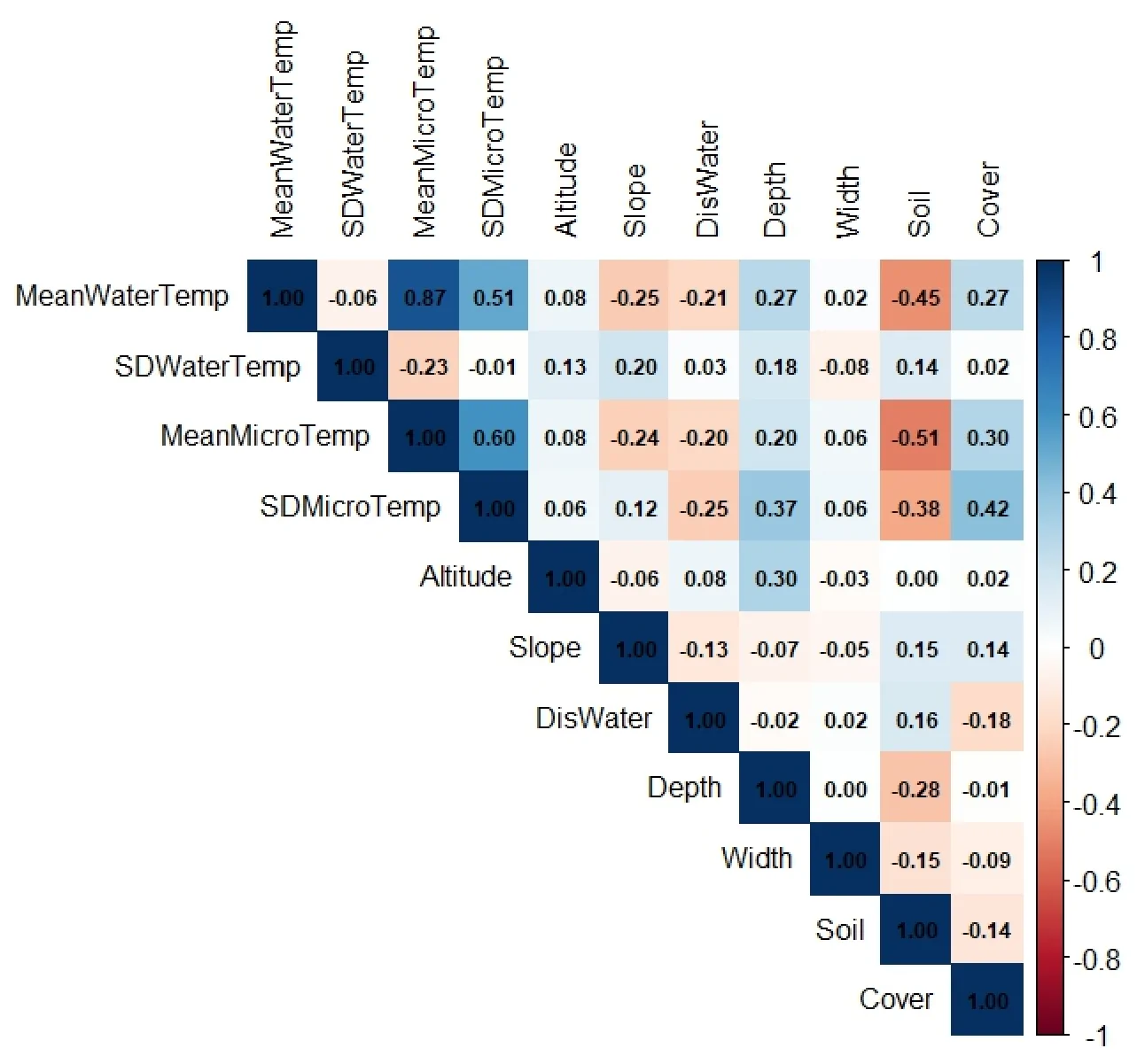
Spearman rank-correlation matrix for continuous environmental variables measured at habitat plots. Correlation coefficients (ρ) indicate the direction and strength of pairwise associations.

Spearman’s rank correlation analysis showed that Mean WaterTemp was strongly positively correlated with Mean MicroTemp (ρ = 0.865, *p* < 0.001; Supplementary Table S1), whereas correlations among the remaining continuous environmental variables were generally weak to moderate (Figure 3). To avoid including highly redundant temperature predictors, Mean MicroTemp was excluded from subsequent candidate models, whereas Mean WaterTemp was retained as an aquatic predictor. Multicollinearity diagnostics indicated no problematic collinearity within the aquatic, terrestrial, or integrated model sets, with adjusted GVIF values ranging from 1.03 to 2.58 (Supplementary Table S2).

### 3.3. Model Performance

Three model structures representing aquatic, terrestrial, and integrated environmental characteristics were evaluated to examine habitat use by EWS. Candidate-model construction and the number of models evaluated within the aquatic and terrestrial model sets are summarized in Supplementary Table S3.

#### 3.3.1. Aquatic Habitat-Use Model

The global aquatic habitat model included season and six aquatic environmental variables: Mean WaterTemp, water-temperature variability, DisWater, Velocity, Depth, and Width. Model selection based on the corrected Akaike’s Information Criterion (AICc) identified four competing models with substantial support (ΔAICc < 2; Supplementary Table S4), indicating some uncertainty in the selection of a single best model. Therefore, model averaging across the four supported models was used to account for model-selection uncertainty.

DisWater and Velocity received the strongest support among aquatic habitat variables, with summed Akaike weights (Σw) of 1.00 for both predictors (Supplementary Table S5). Model-averaged estimates showed that habitat use decreased significantly with increasing distance from water (β = −0.319, *p* = 0.004). The corresponding odds ratio was 0.73, indicating that the odds of habitat use decreased by approximately 27% for each 1 m increase in distance from water. Velocity also showed strong support: EWS were more likely to use still-water habitats than fast-flowing habitats (β = 1.900, *p* = 0.013), whereas no significant difference was detected between slow- and fast-flowing habitats (*p* = 0.376).

Mean WaterTemp received moderate model support (Σw = 0.59), but its model-averaged effect was uncertain (β = 0.136, *p* = 0.388). Width received weaker support (Σw = 0.35) and showed no clear association with habitat use. Overall, proximity to water and water-flow conditions represented the most consistently supported aquatic characteristics associated with EWS habitat use (Table 4).

#### 3.3.2. Terrestrial Habitat-Use Model

The global terrestrial habitat model included season and ten terrestrial environmental variables describing microclimate, topography, substrate conditions, vegetation structure, and Human. AICc-based model selection produced considerable model-selection uncertainty, with 25 models falling within ΔAICc < 2. Accordingly, inference was based on full model averaging rather than selection of a single terrestrial model.

SP received the strongest support among terrestrial habitat predictors (Σw = 1.00), followed by SD MicroTemp (Σw = 0.86) and soil (Σw = 0.61; Table S5). EWS were significantly less likely to use upper-slope than lower-slope sites (β = −2.61, *p* = 0.023), whereas middle- and lower-slope sites did not differ significantly. SD MicroTemp received relatively strong model support, although its full model-averaged coefficient remained uncertain (β = 0.32, *p* = 0.136). Other terrestrial predictors received comparatively weaker support across the candidate-model set (Table 5; Supplementary Tables S5 and S6).

#### 3.3.3. Integrated Habitat-Use Model and Model Comparison

Predictors receiving consistently strong support in the component-specific multimodel analyses (Σw ≥ 0.80) were carried forward to the integrated habitat model. The resulting model included DisWater, Velocity, SP, and SD MicroTemp, with season retained as an a priori temporal covariate. In the final integrated model, habitat use decreased significantly with increasing distance from water (β = −0.333, *p* = 0.008). EWS were more likely to use still-water habitats than fast-flowing habitats (β = 1.728, *p* = 0.04), whereas slow- and fast-flowing habitats did not differ significantly. Upper-slope sites had substantially lower odds of habitat use than lower-slope sites (β = −2.712, *p* = 0.02). SD MicroTemp showed a positive but statistically uncertain association (β = 0.332, *p* = 0.062). No statistically significant association between season and habitat use was detected in the integrated model (*p* = 0.349; Table 6).

The integrated model received the strongest overall support, with the lowest AICc (101.62), compared with the aquatic (AICc = 106.21, ΔAICc = 4.59) and terrestrial models (AICc = 110.50, ΔAICc = 8.88). Apparent AUC was highest for the integrated model (0.853, 95% bootstrap CI = 0.767–0.925), followed by the terrestrial (0.796, 95% CI = 0.700–0.881) and aquatic models (0.794, 95% CI = 0.694–0.879; Figure S1). This pattern was retained under repeated stratified 10-fold cross-validation, with mean AUC values of 0.786 ± 0.013, 0.737 ± 0.014, and 0.733 ± 0.013 for the integrated, terrestrial, and aquatic models, respectively (Supplementary Table S7 and Figure S2). Five-fold spatially grouped cross-validation of the integrated model yielded a pooled out-of-fold AUC of 0.783, indicating little reduction in predictive discrimination when geographically clustered observations were retained within the same validation group. However, because the original pair identifiers were unavailable, this analysis does not constitute pair-preserving cross-validation. Overall, the integrated predictor set provided stronger model support and higher discriminatory performance in this dataset than either the aquatic or terrestrial predictor set alone.

The principal associations with distance to water, water velocity, and slope position were broadly consistent when the integrated model was expanded beyond the original Σw ≥ 0.80 inclusion threshold. Adding the next-most-supported aquatic and terrestrial predictors separately or jointly produced similar effect estimates for these three predictors (Supplementary Table S8). The expanded models had AICc values of 102.67, 103.26, and 104.52, respectively, compared with 101.62 for the original integrated model. Across all expanded models, the negative association with DisWater, the positive association with still versus fast-flowing water, and the negative association with upper versus lower slope position retained the same direction and statistical support. Thus, these three principal habitat-use associations were not strongly sensitive to the specific Σw ≥ 0.80 threshold used to construct the integrated model. Diagnostic and sensitivity analyses indicated that the principal associations with distance to water and water velocity were not driven by any single observation (Supplementary Table S9). A comparison of model support and discriminatory performance among the three model structures is presented in Table 7.

**Figure 4:**
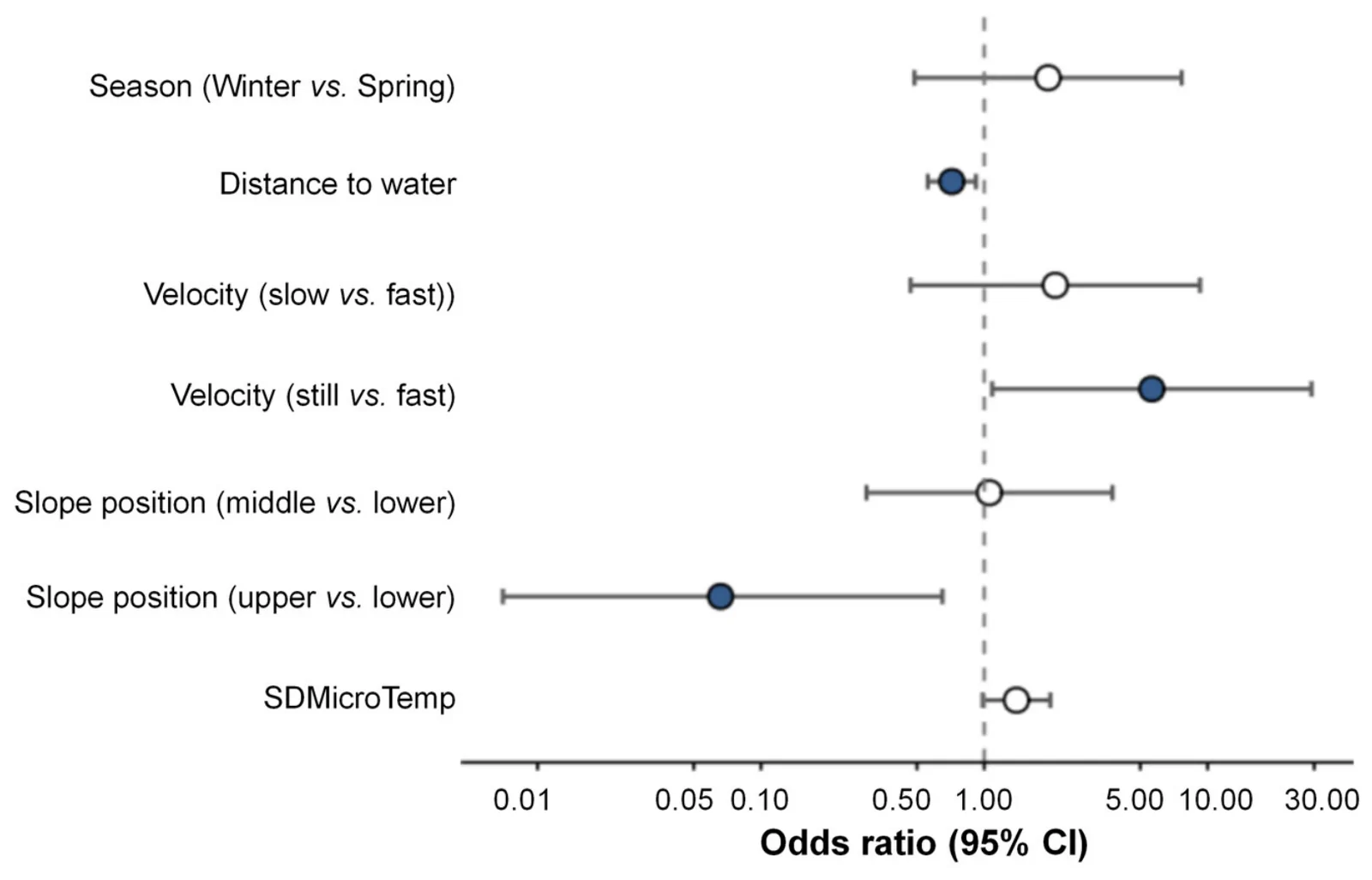
Effect sizes of predictors included in the integrated habitat-use model for EWS. Points represent odds ratios (ORs), and horizontal lines indicate 95% confidence intervals. Filled points denote effects with *p* < 0.05. The vertical dashed line represents OR = 1. Spring, fast-flowing water, and lower-slope position were the reference categories for Season, Velocity, and slope position (SP), respectively.

**Figure 5:**
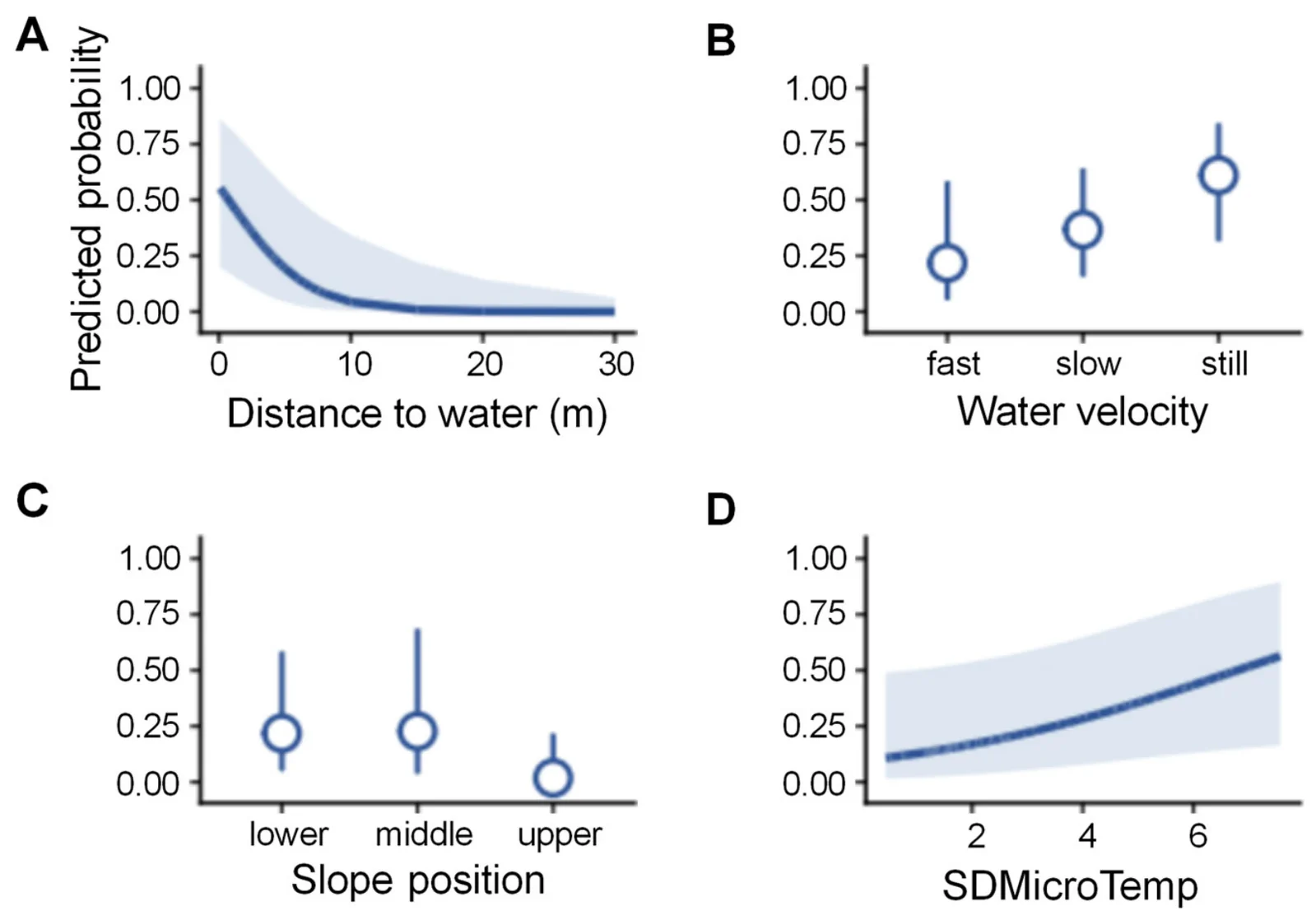
Model-adjusted relationships between principal environmental predictors and the probability of habitat use by Eurasian water shrew (EWS). Predicted probabilities were derived from the final integrated logistic regression model. (**A**) Distance to water (DisWater), (**B**) water velocity (Velocity), (**C**) slope position (SP), and (**D**) standard deviation of microhabitat temperature (SD MicroTemp). Lines or points represent model-predicted probabilities, and shaded bands or error bars indicate 95% confidence intervals. Predictions for each focal predictor were generated while accounting for the remaining predictors in the integrated model.

Residual spatial autocorrelation in the integrated GLM was weak and sensitive to neighborhood definition. Moran’s I was 0.108 (permutation *p* = 0.048) using four nearest neighbors, whereas statistical support for residual spatial autocorrelation was not detected using six (I = 0.045, *p* = 0.152) or eight nearest neighbors (I = 0.040, *p* = 0.139). In the spatial GAM, the two-dimensional spatial smooth was not statistically supported (*p* = 0.321), while the direction and statistical support of the principal habitat-use associations were broadly consistent with those of the integrated GLM. DisWater remained negatively associated with habitat use (β = −0.366, *p* = 0.005), still-water habitats remained positively associated with habitat use relative to fast-flowing habitats (β = 1.745, *p* = 0.043), and upper-slope sites remained negatively associated with habitat use relative to lower-slope sites (β = −2.848, *p* = 0.017; Supplementary Table S10). Residual spatial autocorrelation was no longer significant after spatial adjustment across the three neighborhood definitions (Supplementary Table S11). The effect sizes of the predictors included in the integrated model are shown in Figure 4. Model-adjusted predicted probabilities for the principal predictors are shown in Figure 5.

## 4. Discussion

Ecological information on EWS remains limited in China, where available records are primarily concentrated in northeastern forest ecosystems. Their cryptic behavior and close association with riparian environments make field-based ecological studies particularly challenging. By jointly evaluating aquatic and terrestrial environmental characteristics, the present study provides quantitative evidence of associations between environmental characteristics and EWS habitat use. The results highlight the importance of considering both components of the aquatic–terrestrial interface when evaluating habitat use by this semi-aquatic species.

### 4.1. Integrated Aquatic–Terrestrial Characteristics Better Characterize EWS Habitat Use

The integrated model received the strongest overall support among the three habitat-use models, with the lowest AICc (101.62), compared with the aquatic and terrestrial models. The same pattern was observed for discriminatory performance. The integrated model had the highest apparent AUC (0.853). This advantage was retained under repeated stratified 10-fold cross-validation. These results provide consistent evidence that considering aquatic and terrestrial environmental characteristics jointly better characterized EWS habitat use than considering either habitat component separately. However, these model comparisons quantify differences in model support and discriminatory performance and should not be interpreted as evidence of independent or causal contributions of aquatic and terrestrial habitat components. Nevertheless, the integrated model was constructed using a two-stage, data-dependent screening procedure, and the expanded-model sensitivity analysis does not fully propagate uncertainty from the initial component-specific model-selection step. The integrated-model results should therefore be interpreted as exploratory rather than confirmatory.

This pattern is consistent with the semi-aquatic ecology of EWS. Previous studies have shown that EWS use both aquatic environments and adjacent terrestrial habitats [28]. Greenwood et al. [15] identified several characteristics of streams and their margins associated with water-shrew occurrence, including current speed, depth, bank incline, and bank-side vegetation. More recently, van der Putten et al. [17] demonstrated through radio tracking and LiDAR-based habitat characterization that fine-scale habitat use by EWS extended across aquatic and terrestrial environments. These findings support the view that habitat use by EWS cannot be fully represented by characteristics of the water body alone.

The improvement in model performance is also important from a predictive perspective. The higher repeated cross-validated AUC of the integrated model indicates that its advantage was retained when predictions were evaluated on held-out observations, rather than being restricted to apparent discrimination based on the data used for model fitting. Nevertheless, the cross-validated AUC of 0.786 indicates good rather than complete discrimination. The integrated model should therefore be interpreted as providing a better characterization of habitat use relative to the two component-specific models, rather than as capturing all environmental characteristics associated with EWS habitat use. Accordingly, inference from the integrated model should be regarded as exploratory, because the two-stage model-building procedure does not fully propagate uncertainty from the initial aquatic and terrestrial model-selection steps.

### 4.2. DisWater and Water Velocity Characterize Aquatic Habitat Use

DisWater was consistently associated with EWS habitat use. In the integrated model, habitat use decreased significantly with increasing distance from water (β = −0.333, *p* = 0.008). This result is consistent with the close association between EWS and aquatic environments reported in previous studies. In particular, van der Putten et al. [17] found that the probability of EWS occurrence declined with increasing distance from water based on fine-scale radio-tracking data. Our findings provide comparable evidence from the Lesser Khingan Mountains and indicate that proximity to water is an important characteristic of habitat use in this population.

The importance of proximity to water is also consistent with the ecological characteristics of EWS. Previous studies have documented extensive use of aquatic environments by the species and have shown that aquatic prey constitute an important component of its diet [14]. However, the present study did not quantify prey availability, foraging success, or individual movement. Therefore, although several mechanisms could potentially contribute to the observed association with DisWater, our results specifically support an association between DisWater and habitat use, rather than a particular underlying mechanism. Water velocity was another aquatic characteristic associated with habitat use. In the integrated model, EWS were more likely to use still-water habitats than fast-flowing habitats (β = 1.728, *p* = 0.040), whereas no statistically significant difference was detected between slow- and fast-flowing habitats. This association indicates higher use of still-water habitats under the environmental conditions represented by our sampling sites.

Several ecological mechanisms could potentially contribute to this association. According to optimal foraging theory, animals are expected to balance energetic gains against the costs of resource acquisition [29]. Given the high metabolic demands of shrews [30], reduced energetic costs during aquatic foraging represent one possible hypothesis for the observed association with still- or slow-flowing water. However, prey availability, foraging success, energetic expenditure, and individual movement were not measured in the present study. These mechanisms therefore remain hypotheses rather than explanations directly supported by our data.

The relationship between water velocity and EWS habitat use does not appear to be uniform among previous studies. Greenwood et al. [15], for example, reported an association between water-shrew occurrence and fast-flowing, relatively shallow water in southeastern England, which differs from the higher use of still-water habitats observed in our study. Accordingly, our result should not be interpreted as evidence of a universal preference for still water, but rather as an association under the environmental conditions and spatial scale examined in the Lesser Khingan Mountains. Interpretation of the still-water association should also account for the qualitative nature of the water-velocity classification, because no instrumental current-velocity measurements or formal observer-repeatability assessments were available. Further comparative studies incorporating quantitative prey measurements and individual movement data would be required to determine the mechanisms underlying variation in water-velocity associations among populations and regions.

### 4.3. Slope Position Was Associated with Terrestrial Habitat Use

Among the terrestrial characteristics retained in the integrated model, slope position was associated with habitat use. EWS showed lower habitat use at upper-slope than at lower-slope sites (β = −2.712, *p* = 0.020). This result suggests that terrestrial topographic position may also be relevant to EWS habitat use, although estimates for sparsely represented slope-position categories should be interpreted cautiously.

Previous studies provide some evidence that topographic characteristics of stream margins are relevant to EWS occurrence. Greenwood et al. [15] identified bank incline among the habitat characteristics associated with water-shrew occurrence in southeastern England. However, bank incline and the slope-position categories used in the present study represent different environmental measurements and should not be treated as directly equivalent. In the present dataset, habitat use was lower at upper-slope sites than at lower-slope sites.

The inclusion of slope position alongside DisWater and water velocity in the integrated model reinforces the value of considering terrestrial characteristics together with aquatic characteristics when characterizing EWS habitat use. For a semi-aquatic species occupying the interface between streams and surrounding terrestrial environments, consideration of both habitat components is therefore important when characterizing habitat use.

### 4.4. Ecological and Conservation Implications and Study Limitations

The combined results identify three environmental characteristics with clear associations with EWS habitat use in the Lesser Khingan Mountains: DisWater, water velocity, and slope position. Habitat use decreased with increasing distance from water, was higher in still-water than in fast-flowing habitats, and was lower at upper-slope than at lower-slope sites. Together with the stronger support and higher discriminatory performance of the integrated model, these findings indicate that habitat assessments for EWS should consider both aquatic conditions and characteristics of the adjacent terrestrial environment.

From a conservation perspective, the results support maintaining the integrity of the aquatic–terrestrial interface in habitats occupied by EWS. In particular, conservation assessments should not focus exclusively on conditions within streams while overlooking the surrounding terrestrial environment. Maintaining heterogeneous stream-margin environments that include aquatic habitats and adjacent lower-slope areas may help preserve the range of habitat conditions associated with EWS occurrence in the study area. However, the observed higher use of still-water habitats should not be translated directly into a universal flow-management recommendation. Previous studies have reported different relationships between water velocity and EWS occurrence [15], and our results apply specifically to the environmental conditions represented by the Lesser Khingan Mountains.

Several limitations define the scope of these conclusions. First, the study was conducted during winter and spring in a single region of northeastern China. The observed habitat associations therefore require further evaluation during other periods of the annual cycle and in other parts of the species’ range. Second, habitat characteristics were quantified at the sampling-plot scale, whereas individual movement, home-range structure, and broader landscape connectivity were not measured. Third, prey availability was not quantified. Consequently, the associations between habitat use, DisWater, and water velocity cannot be interpreted directly in terms of food availability or foraging efficiency [31]. Although the integrated model showed the strongest support and the highest discriminatory performance, its repeated cross-validated AUC of 0.786 indicates that habitat use was not completely explained by the variables included in the model. Previous studies have shown that soil physicochemical characteristics, particularly pH and salinity, can be associated with riparian plant-community composition and diversity [32]. These factors may influence riparian vegetation and thereby contribute indirectly to habitat conditions available to EWS; their potential contribution should therefore be evaluated explicitly in future studies.

An important limitation concerns the original used–control sampling structure. Although field sampling followed a 1:1 allocation of used and control plots, a reliable plot-level pairing identifier was not retained in the archived analytical dataset. We therefore could not fit a conditional logistic regression or a model including pair-specific random effects. The spatial GAM and spatially grouped cross-validation evaluate geographic dependence but cannot substitute for explicit modeling of the original matched design. Consequently, residual dependence between originally associated used and control plots cannot be excluded, and the uncertainty of the GLM coefficients may therefore be underestimated. The reported effect estimates and *p* values should thus be interpreted as exploratory plot-scale associations. Future studies should retain explicit pair identifiers or other grouping information at the time of field sampling so that the matched sampling structure can be incorporated directly into statistical analysis.

The spatial analyses also indicate that some caution is warranted regarding the spatial structure of the sampling locations. Residual spatial autocorrelation in the integrated GLM was weak and dependent on neighborhood definition, with statistical support detected only under the four-nearest-neighbor specification. When geographic spatial structure was incorporated explicitly using a two-dimensional smooth in the spatial GAM, the principal habitat-use associations remained broadly consistent with those of the integrated GLM, and residual spatial autocorrelation was reduced. Predictive discrimination was also very similar between repeated stratified cross-validation (AUC = 0.786 ± 0.013) and five-fold spatially grouped cross-validation (AUC = 0.783), indicating little reduction in discrimination when geographically clustered observations were retained within the same validation group. These sensitivity analyses suggest that the principal results were not substantially altered by the spatial structure represented in the present dataset. Nevertheless, spatial dependence at scales not captured by the current sampling configuration cannot be excluded, and the modest sample size and spatial distribution of the plots should be considered when extrapolating these habitat-use associations beyond the surveyed locations. Given the moderate sample size relative to the initial number of candidate predictors, coefficient estimates—particularly for sparsely represented categorical levels—should be interpreted cautiously despite the model-selection, influence, and sensitivity analyses conducted.

In addition, the 10 × 10 m sampling plots were designed to characterize local habitat conditions associated with EWS occurrence and therefore do not resolve habitat selection at finer behavioral or movement scales. Several potentially relevant fine-scale habitat features, including bank structure, refuge availability, woody debris, and detailed riparian vegetation architecture, were not quantified. An additional limitation concerns imperfect detection. Used plots were identified from direct observations and diagnostic field signs, whereas control plots were defined by the absence of detected diagnostic evidence during the corresponding survey. In addition, different types of diagnostic evidence may reflect different forms of EWS activity and therefore cannot be used to distinguish specific behavioral states or the intensity of habitat use.

Although the same field team and standardized survey procedure were used throughout the study, repeated detection–nondetection surveys were not conducted and plot-level search duration was not recorded quantitatively. Detection probability therefore could not be estimated retrospectively, and heterogeneity in detection among plots cannot be excluded. Some control plots may consequently have represented locations used by EWS but not detected during the survey. Such false-negative classification could reduce the observed contrast between used and control plots and introduce uncertainty into the estimated habitat associations. Accordingly, our results should be interpreted as plot-scale habitat associations rather than a complete characterization of fine-scale habitat selection. Future studies incorporating repeated detection surveys, fine-scale microhabitat measurements, prey availability, and individual movement data would allow EWS habitat use and selection to be evaluated more comprehensively across multiple spatial scales.

Overall, our results indicate that EWS habitat use in the Lesser Khingan Mountains was associated with characteristics of both aquatic and terrestrial environments. The stronger model support and higher discriminatory performance of the integrated model further indicate that, within the present dataset, considering aquatic and terrestrial characteristics jointly provided a better characterization of EWS habitat use than considering either habitat component separately.

## 5. Conclusions

This study provides a quantitative assessment of Eurasian water shrew (*Neomys fodiens*, EWS) habitat use in the Lesser Khingan Mountains of northeastern China using an integrated aquatic–terrestrial framework. Distance to water and water velocity were associated with EWS habitat use within the surveyed plots, with habitat use decreasing with increasing distance from water and being higher in still-water than in fast-flowing habitats. Slope position was also associated with habitat use, with lower use of upper-slope than lower-slope sites. Among the three model structures evaluated, the integrated model received the strongest support and showed the highest discriminatory performance, indicating that EWS habitat use in the present dataset was better characterized by considering aquatic and terrestrial environmental characteristics jointly rather than separately. These findings highlight the importance of the aquatic–terrestrial interface for understanding EWS habitat use in the Lesser Khingan Mountains.

## Figures and Tables

**Table 1:** Habitat component measured at used and control plots of EWS.

Category	Habitat Factors	Description	Abbreviation
Aquatic environmental variables	Distance to water (m)	Shortest distance between the plot center and the nearest water body.	DisWater
	Water depth (m)	Average depth measured at three representative locations.	Depth
	Stream width (m)	Mean stream width measured at three representative points within each sampling plot.	Width
	Water velocity	Overall water-flow condition adjacent to the sampling plot, visually classified as fast, slow, or still.	Velocity
	Mean water temperature (°C)	Mean water temperature recorded continuously at 1 h intervals over three consecutive days.	Mean WaterTemp
	Water temperature variability (°C)	Standard deviation of water temperature over the monitoring period.	SD WaterTemp
Terrestrial environmental variables	Altitude (m)	Elevation above sea level measured using GPS.	Altitude
	Slope degree (°)	Measured by the military compass Type 65.	Slope
	Aspect type	Measured in the counterclockwise direction by the military compass Type 65 with the true north direction at 0°. Sunny slope (157.5–337.5°), Shady slope (337.5–157.5°).	Aspect type
	Slope position	Relative position on the hillside classified as upper, middle, or lower slope.	SP
	Human disturbance	Human disturbance was classified into three levels based on the presence and intensity of anthropogenic activities, including roads, footpaths, logging traces, and recreational use. Low disturbance indicated little or no visible human activity, whereas high disturbance indicated frequent or obvious anthropogenic influence.	Human
	Soil (m)	Thickness of the surface soil layer measured using a soil probe.	Soil
	Vegetation cover (proportion)	Vegetation cover was estimated using five 1 m × 1 m quadrats positioned at the center and four corners of each 10 m × 10 m used or control plot. The mean cover across the five quadrats was expressed as a proportion (0–1) and used in subsequent analyses.	Cover
	Vegetation type	Dominant riparian vegetation surrounding each sampling plot was classified according to vegetation physiognomy into three categories, mixed forest, riparian and shrub.	Vegetation
	Riverbank substrate type	Dominant riverbank substrate was classified into three categories, coarse, fine and organic.	Substrate
	Mean microhabitat temperature (°C)	Mean terrestrial microhabitat temperature recorded continuously at 1 h intervals over three consecutive days.	Mean MicroTemp
	Microhabitat temperature variability (°C)	Standard deviation of terrestrial microhabitat temperature over the monitoring period.	SD MicroTemp

**Table 2:** Comparison of continuous environmental variables between used and control plots.

Variable	Used (Mean ± SD)	Control (Mean ± SD)	W	*p*
Mean WaterTemp (°C)	3.93 ± 3.54	3.03 ± 4.00	831.0	0.255
Water temperature SD (°C)	0.67 ± 0.63	0.82 ± 0.72	1183.5	0.073
Mean MicroTemp (°C)	−0.32 ± 15.57	−1.67 ± 15.86	855.0	0.348
SD MicroTemp (°C)	3.53 ± 2.00	2.37 ± 1.61	656.5	**0.009**
Altitude (m)	306.7 ± 18.2	308.1 ± 12.0	988.5	0.867
Slope (°)	22.5 ± 15.9	25.6 ± 19.8	1107.0	0.243
DisWater (m)	3.36 ± 2.86	5.78 ± 6.44	1359.0	**0.001**
Depth (m)	1.31 ± 1.28	1.21 ± 1.23	936.5	0.796
Width (m)	5.86 ± 4.84	5.18 ± 4.47	829.5	0.249
Soil (m)	0.06 ± 0.05	0.11 ± 0.18	1019.0	0.672
Cover	0.31 ± 0.20	0.17 ± 0.17	676.5	**0.014**

Note: Bold *p* values indicate statistically significant differences (*p* < 0.05).

**Table 3:** Seasonal differences in environmental characteristics between spring and winter sampling sites.

Variable	Spring (Mean ± SD)	Winter (Mean ± SD)	W	*p*
Mean WaterTemp (°C)	6.70 ± 1.75	0.25 ± 2.16	1877	<0.001
SD WaterTemp (°C)	0.50 ± 0.35	0.99 ± 0.82	594	0.002
Mean MicroTemp (°C)	13.75 ± 5.91	−15.74 ± 3.72	1936	<0.001
SD MicroTemp (°C)	3.99 ± 2.11	1.92 ± 0.85	1470	<0.001
Altitude (m)	305.87 ± 20.30	308.91 ± 7.59	1086	0.327
Slope (°)	21.82 ± 19.41	26.25 ± 16.22	764	0.086
DisWater (m)	4.77 ± 6.21	4.37 ± 3.70	761	0.084
Depth (m)	1.37 ± 1.28	1.15 ± 1.22	1132.5	0.171
Width (m)	6.17 ± 5.62	4.87 ± 3.35	1052.5	0.483
Soil (m)	0.03 ± 0.02	0.15 ± 0.18	429.5	<0.001
Cover	0.31 ± 0.23	0.17 ± 0.12	1281.5	0.008

**Table 4:** Model-averaged parameter estimates for aquatic environmental variables associated with Eurasian water shrew (EWS) habitat use.

Variable	β	SE	95% CI	OR	*p*	Σw
DisWater	−0.319	0.111	−0.54–−0.10	0.73	**0.004**	**1.00**
Velocity	—	—	—	—	—	**1.00**
Slow vs. fast	0.611	0.691	−0.743–1.965	1.842	0.376	—
Still vs. fast	1.900	0.767	0.396–3.404	6.688	**0.013**	—
Mean WaterTemp (°C)	0.136	0.158	−0.173–0.445	1.146	0.388	0.59
Width (m)	0.022	0.049	−0.074–0.119	1.023	0.651	0.35

Note: Bold values indicate statistically significant associations (*p* < 0.05) bold Σw values indicate the highest summed Akaike weight. β, regression coefficient; SE, standard error; OR, odds ratio; CI, confidence interval; Σw, summed Akaike weight. Season was retained as an a priori temporal covariate in all candidate models and was therefore not interpreted using Σw. Fast-flowing water was used as the reference category for Velocity.

**Table 5:** Model-averaged parameter estimates for terrestrial environmental variables associated with Eurasian water shrew habitat use.

Variable	β	SE	OR	95% CI	*p*	Σw
SP	—	—	—	—	—	**1.00**
Middle vs. lower	0.431	0.631	1.54	−0.805–1.667	0.494	—
Upper vs. lower	−2.613	1.152	0.07	−4.870–−0.356	**0.023**	—
SDMicroTemp	0.317	0.212	1.37	−0.100–0.733	0.136	**0.86**
Soil	−2.210	2.602	0.11	−7.311–2.891	0.396	0.61
Vegetation	—	—	—	—	—	0.38
Cover	0.899	1.638	2.46	−2.311–4.109	0.583	0.34
Substrate	—	—	—	—	—	0.17

Note: Bold values indicate statistically significant associations (*p* < 0.05); bold Σw values indicate the highest summed Akaike weight.

**Table 6:** Logistic regression estimates for the integrated habitat-use model of Eurasian water shrew (EWS).

Variable	β	SE	OR	95% CI	*p*
Season (Winter vs. Spring)	0.658	0.703	1.931	0.487–7.654	0.349
DisWater	−0.333	0.126	0.716	0.559–0.918	**0.008**
Velocity (slow vs. fast)	0.733	0.761	2.080	0.468–9.251	0.336
Velocity (still vs. fast)	1.728	0.840	5.628	1.085–29.186	**0.040**
SP (middle vs. lower)	0.054	0.647	1.055	0.297–3.751	0.934
SP (upper vs. lower)	−2.712	1.164	0.066	0.007–0.650	**0.020**
SDMicroTemp	0.332	0.178	1.394	0.984–1.976	0.062

Note: Bold *p* values indicate statistically significant associations (*p* < 0.05).

**Table 7:** Comparison of model support and discriminatory performance among aquatic, terrestrial, and integrated habitat-use models.

Model	Model Predictors	AICc	ΔAICc	Apparent AUC (95% Bootstrap CI)	Repeated 10-Fold CV AUC
Aquatic	Season, DisWater, MeanWaterTemp, Velocity	106.21	4.59	0.794 (0.694–0.879)	0.733 ± 0.013
Terrestrial	Season, SDMicroTemp, SP, Soil	110.50	8.88	0.796 (0.700–0.881)	0.737 ± 0.014
Integrated	Season, DisWater, Velocity, SP, SDMicroTemp	101.62	0.00	0.853 (0.767–0.925)	0.786 ± 0.013

Note: For the aquatic and terrestrial components, AICc and predictive performance are reported for the top-ranked model from each component-specific candidate set.

## Data Availability

The original contributions presented in this study are included in the article/Supplementary Material. Further inquiries can be directed to the corresponding authors.

## Supplementary Materials

The following supporting information can be downloaded at: https://www.mdpi.com/article/10.3390/biology15181674/s1, Figure S1: Receiver operating characteristic (ROC) curves for the aquatic, terrestrial, and integrated habitat-use models of Eurasian water shrews; Figure S2: Distribution of cross-validated area under the receiver operating characteristic curve (AUC) for the aquatic, terrestrial, and integrated habitat-use models; Table S1: Spearman rank correlations among continuous environmental variables measured at Eurasian water shrew habitat plots; Tables S2–S11: Multicollinearity diagnostics, candidate-model construction and model-averaging results, cross-validation performance, predictor-inclusion and leave-one-out sensitivity analyses, and spatial analyses.
