# Supplementary material for: Aquatic and Terrestrial Environmental Correlates of Eurasian Water Shrew (Neomys fodiens) Habitat Use in the Lesser Khingan Mountains
Source: Biology (Basel). 2026 Sep 21;15(18):1674. doi: 10.3390/biology15181674 (PMC13604908; doi:10.3390/biology15181674)
Supplement: Supplementary file 1 [file biology-15-01674-s001.zip › biology-4549610-supplementary.pdf]

Table S1. Spearman rank correlations among continuous environmental variables measured at Eurasian water shrew habitat plots.

Variable	1	2	3	4	5	6	7	8	9	10	11
**1. Mean water temperature**	—										
**2. SD water temperature**	-0.062	—									
**3. Mean microhabitat temperature**	**0.865*****	**-0.229***	—								
**4. SD microhabitat temperature**	**0.511*****	-0.008	**0.603*****	—							
**5. Altitude**	0.076	0.129	0.078	0.056	—						
**6. Slope**	**-0.251***	0.202	-0.236*	0.116	-0.062	—					
**7. Distance to water**	-0.210	0.026	-0.202	**-0.246***	0.078	-0.126	—				
**8. Water depth**	**0.273***	0.175	0.198	**0.374*****	**0.304****	-0.069	-0.025	—			
**9. Stream width**	0.023	-0.079	0.059	0.063	-0.030	-0.048	0.017	0.003	—		
**10. Soil surface thickness**	**-0.455*****	0.143	**-0.505*****	**-0.383*****	0.001	0.145	0.160	**-0.285****	-0.152	—	
**11. Vegetation cover**	0.268*	0.018	0.296**	**0.420*****	0.016	0.141	-0.182	-0.006	-0.086	-0.136	—

Note: Values are Spearman's rank correlation coefficients. $P < 0.05$*, $P < 0.01$**, $P < 0.001$***.

Table S2. Multicollinearity diagnostics for the aquatic, terrestrial, and integrated habitat-use models

Modle	Predictor	GVIF	Df	GVIF^(1/(2×Df))
Aquatic	**Season**	6.638	1	**2.576**
	Mean water temperature	5.594	1	2.365
	SD water temperature	1.650	1	1.284
	Distance to water	1.162	1	1.078
	Water velocity	1.241	2	1.056
	Water depth	1.219	1	1.104
	Stream width	1.075	1	1.037
Terrestrial	**Season**	2.232	1	**1.494**
	SD microhabitat temperature	2.099	1	1.449
	Altitude	1.345	1	1.160
	Slope	1.642	1	1.282
	Aspect type	1.266	1	1.125
	Slope position	1.550	2	1.116
	Human disturbance	1.582	2	1.122
	Substrate	1.940	2	1.180
	Soil surface thickness	1.329	1	1.153
	Vegetation cover	1.663	1	1.290
	Vegetation type	1.712	2	1.144
Integrated	**Season**	1.707	1	**1.306**
	Distance to water	1.157	1	1.075
	Water velocity	1.141	2	1.034
	Slope position	1.207	2	1.048
	SD microhabitat temperature	1.558	1	1.248

Table S3. Summary of candidate-model construction for the aquatic and terrestrial component analyses.

Component	Environmental predictor terms	Fixed term	Candidate models	Selection procedure
Aquatic	6	Season	64	All-subsets AICc
Terrestrial	10	Season	1,024	All-subsets AICc

Note： Candidate models were generated using MuMIn::dredge, with Season retained in every candidate model. Models were ranked using AICc, and models with ΔAICc < 2 were included in model averaging.

Table S4. AICc-supported candidate models for aquatic habitat use by Eurasian water shrews

Model	Predictors	K	logLik	AICc	ΔAICc	Akaike weight

A1	Season, Distance to water, Mean water temperature, Water velocity	6	-46.586	**106.21**	**0.00**	0.379
A2	Season + Distance to water + Water velocity	5	-48.065	106.86	0.65	0.274
A3	Season + Distance to water + Mean water temperature + Water velocity + Stream width	7	-46.012	107.42	1.21	0.207
A4	Season + Distance to water + Water velocity + Stream width	6	-47.577	108.19	1.98	0.141

Note: Only models with ΔAICc < 2 are shown. K represents the number of estimated parameters, including the intercept. logLik, log-likelihood; AICc, Akaike's Information Criterion corrected for small sample size; ΔAICc, difference in AICc relative to the top-ranked model. Akaike weights indicate the relative support for each model within the full candidate-model set.

Table S5. Full model-averaged estimates for aquatic and terrestrial habitat-use models of Eurasian water shrews.

Model	Predictor	β	SE	OR	95% CI	P	Σw
Aquatic	**Distance to water**	-0.319	0.111	0.73	0.58–0.90	**0.004**	1.00
	Mean water temperature	0.136	0.158	1.15	0.84–1.56	0.388	0.59
	Velocity (slow *vs.* fast)	0.611	0.691	1.84	0.48–7.14	0.376	1.00
	**Velocity (still *vs.* fast)**	1.900	0.767	6.69	1.49–30.10	**0.013**	1.00
	Season (Winter *vs.* Spring)	0.981	1.153	2.67	0.28–25.55	0.395	1.00
	Stream width	0.022	0.049	1.02	0.93–1.13	0.651	0.35
Terrestrial	SD microhabitat temperature	0.317	0.212	1.37	0.91–2.08	0.136	0.86
	Slope position (middle *vs.* lower)	0.431	0.631	1.54	0.45–5.30	0.494	1.00
	**Slope position (upper *vs.* lower)**	-2.613	1.152	0.07	0.008–0.700	**0.023**	1.00
	Soil surface thickness	-2.210	2.602	0.11	0.001–18.01	0.396	0.61
	Season (Winter *vs.* Spring)	0.995	0.704	2.71	0.68–10.74	0.157	1.00
	Substrate (fine *vs.* coarse)	0.202	0.521	1.22	0.44–3.39	0.698	0.17
	Substrate (organic *vs.* coarse)	-0.040	0.365	0.96	0.47–1.96	0.913	0.17
	Vegetation (riparian *vs.* mixed forest)	0.482	0.760	1.62	0.37–7.18	0.526	0.38
	Vegetation (shrub *vs.* mixed forest)	0.052	0.488	1.05	0.404–2.74	0.915	0.38

Slope	-0.003	0.009	1.00	0.98–1.02	0.778	0.12
Aspect (sunny *vs.* shady)	0.105	0.356	1.111	0.553–2.233	0.767	0.14
Vegetation cover	0.899	1.638	2.457	0.099–60.895	0.583	0.34
Human disturbance (Level 2 *vs.* Level 1)	-0.034	0.220	0.966	0.627–1.488	0.876	0.07
Human disturbance (Level 3 *vs.* Level 1)	-0.090	0.381	0.914	0.433–1.930	0.814	0.07
Altitude	-0.001	0.006	0.999	0.987–1.011	0.853	0.07

Table S6. Relative importance of terrestrial predictors based on summed Akaike weights across AICc-supported models of Eurasian water shrew habitat use.

**Predictor**	**Σw**	**Models containing predictor (n/25)**
Slope position	**1.00**	25/25
SD microhabitat temperature	**0.86**	21/25
Soil surface thickness	0.61	15/25
Vegetation type	0.38	10/25
Vegetation cover	0.34	10/25
Substrate type	0.17	4/25
Aspect type	0.14	4/25
Slope	0.12	3/25
Human disturbance	0.07	2/25
Altitude	0.07	2/25

Note: Σw denotes the summed Akaike weight across models with ΔAICc < 2. Season was retained as an a priori temporal covariate in all candidate models and was therefore excluded from the predictor-importance comparison.

Table S7. Performance of the aquatic, terrestrial, and integrated habitat-use models based on repeated stratified 10-fold cross-validation.

**Model**	**Mean CV AUC**	**SD**	**Median CV AUC**
Aquatic	0.733	0.013	0.735
Terrestrial	0.737	0.014	0.739
**Integrated**	**0.786**	**0.013**	**0.787**

Note: CV, cross-validation; AUC, area under the receiver operating characteristic curve. Values represent the mean and standard deviation of AUC estimates obtained from 100 repetitions of stratified 10-fold cross-validation. Within each repetition, models were refitted using nine folds and evaluated using predictions for the held-out fold.

Table S8. Sensitivity of the integrated habitat-use model to the predictor-inclusion threshold.

Model	Additional predictor(s)	AICc	ΔAICc	DisWater β (*P*)	Velocity: still *vs.* fast β (*P*)	SP: upper *vs.* lower β (*P*)	SDMicroTemp β (*P*)
Original	None	101.62	0.00	−0.334 (0.008)	1.728 (0.040)	−2.712 (0.020)	0.332 (0.062)
Expanded 1	+ Soil	102.67	1.04	−0.302 (0.015)	1.701 (0.047)	−2.549 (0.029)	0.300 (0.092)
Expanded 2	+ MeanWaterTemp	103.26	1.63	−0.333 (0.008)	1.694 (0.044)	−2.691 (0.023)	0.292 (0.104)
Expanded 3	+ Soil + MeanWaterTemp	104.52	2.90	−0.305 (0.014)	1.681 (0.049)	−2.539 (0.032)	0.266 (0.138)

Note: The original integrated model included Season, DisWater, Velocity, SP, and SD MicroTemp. Expanded models were constructed by adding the next-most-supported predictor from the terrestrial and aquatic component-specific analyses separately and jointly. ΔAICc values are calculated relative to the original integrated model. The expanded models were used as a threshold-sensitivity analysis and were not treated as a new model-selection procedure.

Table S9. Influence diagnostics and leave-one-out sensitivity analysis of the integrated habitat-use model.

Predictor	Primary β	LOO β range	Direction retained	P < 0.05
DisWater	−0.333	−0.375 to −0.305	88/88	88/88
Velocity still *vs.* fast	1.728	1.433–2.284	88/88	79/88
SP upper *vs.* lower	−2.712	−22.782 to −2.373	88/88	87/88
SDMicroTemp	0.332	0.290–0.409	88/88	11/88

Note: Leave-one-out (LOO) sensitivity analysis was conducted by sequentially omitting each observation and refitting the integrated GLM. The upper-slope category contained only one used plot; consequently, omission of this observation resulted in instability of the upper-slope coefficient, and the corresponding LOO range should be interpreted cautiously. Cook's distance and DFBETAs were used as supplementary influence diagnostics and were not used as automatic criteria for observation exclusion.

Table S10. Sensitivity analysis of the integrated habitat-use model after accounting for spatial structure.

Predictor	Primary GLM β	SE	*P*	Spatial GAM β	SE	*P*
Season Winter	0.658	0.703	0.349	0.377	0.760	0.620
DisWater	−0.333	0.126	**0.008**	−0.366	0.132	**0.005**
Velocity slow	0.733	0.761	0.336	0.719	0.776	0.354
Velocity still	1.728	0.840	**0.040**	1.745	0.861	**0.043**
Slope Position middle	0.054	0.647	0.934	−0.113	0.666	0.865
Slope Position upper	−2.712	1.164	**0.020**	−2.848	1.195	**0.017**
SD Micro Temp	0.332	0.178	0.062	0.387	0.186	**0.037**

Table S11. Residual spatial autocorrelation.

Model	k	Moran's I	Permutation P
Primary integrated GLM	4	0.108	**0.048**
Primary integrated GLM	6	0.045	0.152
Primary integrated GLM	8	0.040	0.139
Spatial GAM	4	0.078	0.098
Spatial GAM	6	0.028	0.223
Spatial GAM	8	0.025	0.204

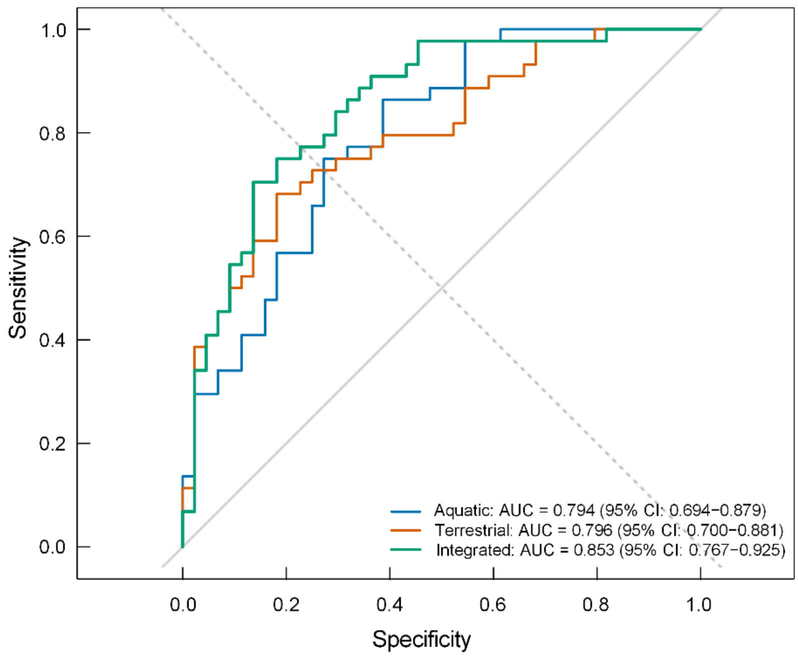

Figure S1. Receiver operating characteristic (ROC) curves for the aquatic, terrestrial, and integrated habitat-use models of Eurasian water shrews. The 95% confidence intervals were estimated using 2,000

stratified bootstrap replicates. The dashed diagonal line represents discrimination expected by chance (AUC = 0.5).

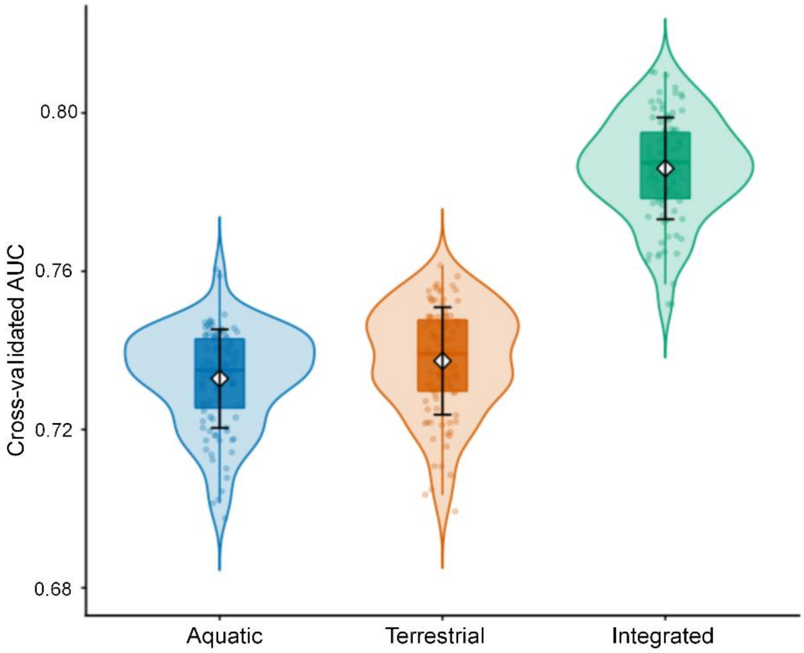

Figure S2. Distribution of cross-validated area under the receiver operating characteristic curve (AUC) for the aquatic, terrestrial, and integrated habitat-use models. AUC values were obtained from 100 repetitions of stratified 10-fold cross-validation. For each repetition, models were refitted using the training folds and predictions were generated for the held-out observations. Boxes indicate the interquartile range, horizontal lines indicate medians, and individual points represent AUC values from each repetition. Diamonds indicate mean AUC values.
